# The Impact of Relative Poverty on Norwegian Adolescents’ Subjective Health: A Causal Analysis with Propensity Score Matching

**DOI:** 10.3390/ijerph9124715

**Published:** 2012-12-18

**Authors:** Jon Ivar Elstad, Axel West Pedersen

**Affiliations:** 1 NOVA-Norwegian Social Research, P.O. Box 3223 Elisenberg, Oslo 0208, Norway; 2 Institute for Social Research, P.O. Box 3233 Elisenberg, Oslo 0208, Norway; E-Mail: axel.w.pedersen@samfunnsforskning.no

**Keywords:** poverty, relative deprivation, adolescence, youth, subjective health, symptoms, causal inference, propensity score matching

## Abstract

Studies have revealed that relative poverty is associated with ill health, but the interpretations of this correlation vary. This article asks whether relative poverty among Norwegian adolescents is causally related to poor subjective health, *i.e.*, self-reported somatic and mental symptoms. Data consist of interview responses from a sample of adolescents (N = 510) and their parents, combined with register data on the family’s economic situation. Relatively poor adolescents had significantly worse subjective health than non-poor adolescents. Relatively poor adolescents also experienced many other social disadvantages, such as parental unemployment and parental ill health. Comparisons between the relatively poor and the non-poor adolescents, using propensity score matching, indicated a negative impact of relative poverty on the subjective health among those adolescents who lived in families with relatively few economic resources. The results suggest that there is a causal component in the association between relative poverty and the symptom burden of disadvantaged adolescents. Relative poverty is only one of many determinants of adolescents’ subjective health, but its role should be acknowledged when policies for promoting adolescent health are designed.

## 1. Introduction

As income inequalities persist, and are even increasing in many countries [1], *relative poverty* affects many families. However, apart from small pockets of dire poverty, relative poverty in affluent countries does not mean absolute deprivation, but merely that the level of economic resources is distinctly lower than the average. In comparison with most other families, relatively poor families will command fewer economic resources and consume fewer goods and services. Relative poverty may nevertheless go hand-in-hand with an access to a material standard of living which, from a historical perspective, is fairly high. This is for instance the case in Norway, the setting of the present study. Using the European Union’s definition of relative poverty, *i.e.*, household-equivalized disposable family income below 60 per cent of the median [2], a 2003 Norwegian survey found that among families who qualified as poor according to this criterion, about 95 per cent had a washing machine, 95 per cent had a deep freezer, and around 80 per cent had a car, a personal computer, and a dishwashing machine. Lack of food and clothes were practically non-existent, and almost all had a fairly suitable dwelling [3].

Should this type of poverty be a health policy concern, in the sense that policy makers should seek a substantial reduction of relative poverty in order to improve population health? An affirmative answer to this question presupposes that relative poverty has a *causal impact* on health. Here this question is examined with respect to adolescents’ health. As serious somatic disease rarely occurs in these age groups, other health-related indicators such as risk behaviours, overweight, and self-rated overall health may be more suitable for examining health inequalities among young people. In this paper the focus is on subjective health, *i.e.*, on self-reported somatic and mental symptoms. We ask: Is living in relative poverty a *cause* of such subjective health complaints among adolescents?

Diverging answers to this question are found in the literature. The *Family Economic Stress Model* [4,5,6] argues that economic hardship will result in family stress due to the mismatch between needs and resources. Family stress will trigger parental conflict, frustration, and hopelessness which affect the lives of the offspring and become manifested in higher levels of subjective health complaints. The *Investment Model* [7] suggests that lack of economic means could lead to a neglect of children’s need for attention and support, because parents have to prioritize immediate material concerns. These two models differ in emphasis, but both assume a causal effect *from* economic resources *to* offspring’s subjective health. This contrasts with accounts which regard associations between family economy and offspring’s developmental outcomes as mostly spurious, a viewpoint forcefully put forward by Mayer’s *What Money Can’t Buy* [8]. In this book, the main narrative is that parents’ personal characteristics which facilitate higher earnings are basically the same traits which create the secure and supporting family environment that promotes good subjective health among children. Thus, there is no important direct causal influence from relative poverty to offspring’s subjective health. If these two are statistically associated, the reason is their joint origin in parental ability and dispositions.

Certainly, both accounts are plausible. Restricted money resources may trigger quarrels between parents and harm family life with detrimental consequences for the offspring’s subjective well-being. Moreover, it is likely that there will always be some parents who both have problematic economic careers and lack the personal qualities which promote subjective health among their children. Individual families exemplifying both patterns will probably be found in any empirical material, and a study of causality will help to establish which pattern predominates in a given population. 

Many empirical studies have demonstrated that families’ relative poverty, as well as parental characteristics such as low education, unskilled occupations and unemployment, tend to be associated with less good health in the offspring. Examples can be found in the United States [9] and Australia [10], as well as in the Nordic countries [6,11,12,13,14,15,16,17,18], Britain [19,20,21,22] and Germany [23,24,25]. Exceptions from the predominant pattern can also be found [26,27] and studies differ considerably with regard to design and variables, but overall, the results indicate higher levels of ill health among adolescents from low income families.

To assess causality from these studies is rather difficult, however, as data well suited for causal analysis are often lacking [28]. With few exceptions [22], only cross-sectional data have been available, making the time-ordering of variables uncertain. Moreover, information about health and socioeconomic status has often been collected by survey answers from only one source—either from parents only, or from children/adolescents only. Therefore, statistical associations may sometimes be artificially produced because of variations in respondents’ response styles; for instance respondents who give negative verdicts of all types of circumstances.

In addition to these difficulties, it is evident that adolescents’ subjective health is influenced by a number of other circumstances apart from the family’s economic situation. A regular finding is for instance that subjective health complaints are more often reported by girls than boys [29]. Individual susceptibility will have a role, as will neighbourhood characteristics and school experiences [30]. Also, the choice of age groups could be consequential. The *equalisation of health in youth* thesis [31,32] suggests that socioeconomic variations in subjective health are practically absent in late childhood and early adolescence, before re-emerging when adolescents approach early adulthood. If so, data covering late adolescence are needed in order to detect a causal effect of relative poverty.

This paper is an attempt to shed further light on these topics. Using longitudinal data material with information collected both from parents, from the adolescents themselves, and from public administrative registers, we make a causal analysis of the association between relative poverty and adolescents’ subjective health, *i.e.*, their reporting of various somatic and mental symptoms. We understand causality in terms of the counterfactual model [33,34,35]. This implies that we seek answers to *what if*-questions: If the relatively poor adolescents had not been poor; would their health have been better? Conversely, as regards the non-poor adolescents; would their health have been worse in the hypothetical case that they lived in relative poverty? *Propensity score matching* is employed in the analyses. As this method, although more and more used in causal analyses [36,37,38], is still not widely known, we first describe shortly some main aspects of this method [34,39,40] before we proceed to data description, analysis, and discussion.

## 2. Causal Analysis by Means of Propensity Score Matching

If we have reasonably valid data about families’ economic situation and the offspring’s subjective health, we can estimate how subjective health differs between adolescents in relatively poor and adolescents in non-poor families. However, this observed difference is likely to be different from a true estimate of the causal effect of relative poverty, because the samples of poor and non-poor families will probably differ not only with respect to economic resources, but also with respect to other circumstances which could be involved in the “production” of subjective health. Thus, the two samples are *imbalanced*: They are differently composed with regard both to their economic situation and to a number of other relevant characteristics.

A solution to this problem is to try to eliminate the imbalance by constructing a *matched* control sample, *i.e.*, to select, in the sample of non-poor adolescents, a subsample which corresponds as closely as possible to the sample of relatively poor adolescents with respect to all relevant circumstances. If this could be done perfectly, we would have two samples which differ only along the poor-non-poor dimension. In other words, we would have approached the balanced composition of the “treatment” group (*i.e.*, those who are exposed to relative poverty) and the “control” group (the non-poor adolescents) which is the aim of randomization. If so, the difference between the relatively poor and the *matched* non-poor adolescents would approximate the true causal effect.

However, all data sets have limitations, and many circumstances, some of them unobserved, are likely to vary between the relatively poor and the non-poor adolescents. Therefore, it is practically impossible to construct a matched sample so that each relatively poor adolescent has a duplicate in the non-poor sample with exactly the same values on all relevant background variables. A way out of this difficulty is to construct a matched sample which is similar to the sample of relatively poor—not with respect to the specific values on a large set of variables, but with respect to the values on a *single* dimension: the *probability* of becoming relatively poor. This probability can be calculated for each individual in the entire sample, both among those who actually became poor and those who avoided poverty. This calculation is usually made by fitting a logit or probit regression model which includes all variables available in the data which could reasonably be considered as being part of, or related to, the trajectories and social processes which lead into relative poverty.

The probability of entering relative poverty, calculated in this way, is termed the *estimated propensity score*. Causal analysis by means of propensity score matching implies that we try to remove the imbalance between the samples by selecting a control sample (here: among those not exposed to poverty) which has the same distribution of estimated propensity scores as the sample who actually was exposed to relative poverty. The method rests on the assumption that a comparison of units with equal propensity scores can effectively substitute for a perfect multidimensional matching on the observed variables [41]. With two samples which have (almost) identical distributions of propensity scores, the desired balance with respect to confounding variables can be achieved, and the difference in outcome will therefore approximate the true causal effect of being exposed to relative poverty.

Thus, matching by estimated propensity scores may remove the imbalance between the two samples with respect to the observed variables. However, the technique does not provide a guarantee against bias arising from unobserved variables [42]. Matching by propensity scores does not avoid the problem posed by unobserved heterogeneity, but the method is a useful tool when we try to estimate causal effects in observational data.

In comparison to regression techniques, matching by estimated propensity scores can have some advantages [34,35]. For instance, the adjustment for confounding factors achieved through conventional multivariate regression will usually rest on assumptions about linearity and additivity. In contrast, matching in general, and propensity score matching in particular, is a non-parametric strategy which can be used without making assumptions about the functional form of the associations, or about the presence of interaction effects between the control variables. Multivariate regression analyses will often lead to inferences based on extrapolation outside the range of the observed data points, while matching by propensity scores can avoid this difficulty. While regression analyses often imply that an explanatory variable has “an invariant, structural causal effect that applies to all members of the population” [34], propensity score matching is a convenient tool for examining how effects differ between various subcategories in the sample.

## 3. Data, Variables, and Statistical Analysis

### 3.1. Data and Analyzed Sample

Data come from the project *Children’s level of living—the impact of family income* conducted at Norwegian Social Research (NOVA) and co-financed by Norwegian Women’s Public Health Association [43,44]. Statistics Norway was commissioned to obtain personal interviews with a sample of families with children, in three interview waves 2003–2009. Information from Statistics Norway’s educational register and taxation register has been linked to the sample by means of the personal identification number. Data were made available for research after being anonymized according to the regulations of the Norwegian Data Inspectorate.

In this study, adolescents born 1991–1993 are analyzed, because this birth cohort was asked about subjective health both in the 2009 interviews when aged 16–18, and in the 2003 interviews when aged 10–12. This enables analyses both of the *level* of subjective health in late adolescence and of *changes* in subjective health during adolescence. As late adolescence often will be a critical period with far-reaching consequences for adult life, a focus on this life phase is particularly interesting. A supplementary reason for examining this age category was that socioeconomic variations in subjective health may be easier to detect among 16–18 years old than among younger adolescents [31]. Altogether, 557 adolescents born in 1991–1993 participated both in the 2003 and 2009 data collection waves. Among them, 510 (91.6 per cent) gave complete answers to the subjective health questions in both years, and these 510 adolescents (268 girls, 242 boys) are analyzed in the present study.

Although random sampling from population registers was utilized in the upstart of the project, the sample analyzed in this paper cannot be considered a representative sample of Norwegian adolescents born in 1991–1993, mainly because of sample attrition. Of those who originally were included in the gross sample, only 38 per cent were interviewed in 2009. Moreover, the sample was first divided into one subsample drawn randomly from the 2000 population register among all families with children, and another larger subsample drawn randomly among families with children who had low incomes in 2000—a particular purpose of the project was to study living conditions among low-income families with children. In the present study, these two subsamples have been pooled, which can be justified as the data collection procedure was identical in the two subsamples. Moreover, upward income mobility has been substantial in the low-income sample, resulting in quite diluted economic differences between the two subsamples in 2009. The topic of the present study is not to make precise estimations of the overall life situation among Norwegian adolescents aged 16–18, but to analyze possible causal effects of relative poverty. This purpose may be obtained fairly well even though the analyzed sample deviates somewhat from a fully representative sample. A population register analysis of all families with children in Norway in 2007 [45] suggests that the sample analyzed in the present study has some low income bias, but higher income strata are well represented as nearly 40 per cent of the sample had equivalized incomes above the national median in 2008.

### 3.2. Measurements of Subjective Health and Relative Poverty

The measurement of subjective health in this study corresponds to the measurement used by the large cross-national project *Health Behaviour in School Children* [17,29]. Answers to eight questions, given in personal interviews, about frequency of headache, stomach pain, back pain, feeling low, bad temper, nervousness, dizziness and sleeplessness were coded from never = 0 to several times a week = 4 and added into an index. The index’ theoretical range is from zero to 32. Two outcomes are analyzed in this study; the *level* of symptom burden in 2009, and the *change* in this burden from 2003 to 2009.

While the health outcome was gauged by survey answers given by the adolescents themselves, the classification of the sample into relatively poor and non-poor was based on tax register data obtained from Statistics Norway. This ensures a more objective assessment of the family’s economic resources than assessments based on subjective survey statements. The tax registers provided information, for each year 2000–2008, about the sum of all family members’ post-tax income, and the sum of financial assets, *i.e.*, the value of bank accounts, shares, and similar assets which swiftly can be converted into cash. Statistics Norway’s registers were utilized in order to obtain information about each adolescent’s family situation and how it changed because of new siblings, divorce, *etc.* In this way, a fairly precise measurement of each family’s economic situation has been obtained for each year in the period under study. For all the 510 adolescents who had answered the questions about subjective health both in 2003 and 2009, the relevant tax register information was available.

The tax register information was utilized for measuring the families’ economic resources in a way which reflects two considerations. First, a family’s *real* economic strength may be inadequately indicated by one-year snapshots or short-term fluctuations from year to year. Therefore, we have constructed a measurement which reflects the *average* economic situation during the years 2004–2008, *i.e.*, the period between the interviews in 2003 and 2009. Second, as economic resources are not only income-related, but depend also on monetary supplies besides income [46,47], the measurement combines income information with financial asset information into one single scale. The measurement was calculated by adding the sum of all family members’ post-tax income and financial assets for each year 2004–2008, dividing each year’s sum by household composition weights (first adult = 1.0, other adults = 0.5, each child younger than 18 = 0.3), making adjustments due to changes in price levels (2007 = 100), and finally averaging the equivalized and price-adjusted measurements across the five years 2004–2008. The mean value (rounded) on this scale was Norwegian Kroner (NOK) 406,000 (SD 473,000); the median was considerably lower (NOK 281,000), indicating the skewness of the distribution of economic resources.

Drawing the line between relative poverty and non-poverty will be somewhat arbitrary. We chose to consider families with less than NOK 200,000 on average 2004–2008 as relatively poor. This “poverty line”, about 70 per cent of the sample’s median, resulted in classifying 109 out of the 510 analyzed adolescents (about 20 per cent) as living in relative poverty during the years prior to 2009.

### 3.3. The Estimation of Propensity Scores

Causal analysis by means of propensity score matching starts with an estimation of propensity scores [34,39]. In the context of this study, this implies calculating the *probability* of being relatively poor in 2004–2008 for each individual adolescent in the sample.

In general, all available variables—“as many predictors as possible” [35]—which could be involved in or associated with the families’ entry into, or avoidance of, relative poverty, should be included in this estimation. The families’ previous economic situation, the parents’ employment activity, parental characteristics such as education and health, family structure, and family size, are examples of variables which are likely to be associated with the probability of being relatively poor.

We included fifteen variables in the estimation of propensity scores. The tax registers provided data about the average economic situation before the first interviewing in 2003. One variable measured the average post-tax family income 2000–2003, and another variable indicated the average level of financial assets 2000–2003. Both variables were adjusted for household composition and recalculated into natural logarithms. Mothers’ and fathers’ educational levels were measured with data from Statistics Norway’s educational register. Variables indicating the interviewed adult’s age, the number of family members in 2003, and age and gender of the adolescent, were constructed by means of data found in the population register. A variable indicating families’ immigrant status was also based on population register information. Immigrant status was categorized as Norwegian/Western or non-Western—the restricted sample size made a more detailed classification of immigrant background unfeasible. The interviews with one of the parents (usually the adolescent’s mother) provided information for constructing indicators about adults’ employment in 2003 and family structure in 2003. The adolescents were classified as living with both parents, with a single parent, or in a stepfamily, *i.e.*, living with one parent and his/her new partner. Variables about overall self-assessed health among the adults in the family in 2003, and the interviewed adult’s mental health in 2003, were also made with information from the 2003 interviews with one of the parents. In the estimation of propensity scores, we also added a variable indicating subsample belonging (cf. Section 3.1). Categorical variables were indicated by dummy variables in the model. Missing values on the variables used for estimating propensity scores were generally few (0.5–4.0 per cent); these were replaced by the sample’s median value in order to preserve all the 510 adolescents for further analyses.

The propensity score estimation was made by a probit regression model (outcome relatively poor = 1, not poor = 0), and the resulting coefficients were recalculated into probabilities assigned to each adolescent in the sample. STATA version 11.2, with the software *psmatch2*, was used for these estimations [40].

### 3.4. Estimating Counterfactual Effects by Radius and Kernel Matching

Estimated propensity scores can be utilized in further analyses in several ways, for instance as a stratifying tool [34] or as a covariate in OLS multivariate regression [35]. In this study, we utilize more advanced techniques: *Radius* matching and *Kernel* matching. These two methods are variants of the matching algorithms included in STATA’s software *psmatch2* and recommended in the literature [34,39,40]. We chose to use two different techniques; our sample is restricted in size and results may be subject to random errors, and alternative estimation techniques may provide a robustness test of the findings. Below we describe a few main aspects of these two techniques.

The assumption underlying propensity score matching is that two samples with identical distributions of estimated propensity scores will be balanced with respect to a number of possible confounders. The matching sample (here: adolescents not exposed to relative poverty) serves as a basis for the estimations of counterfactual effects; the outcome value in the matching sample is likely to indicate what the outcome value *would have been* in the exposed sample, in the hypothetical situation that exposure to the assumed causal factor had not taken place. It is not unproblematic, however, to construct a matching sample. For instance, as the estimated propensity scores, *i.e.*, the probability of becoming relatively poor, constitute a continuous scale, there will often be instances where no exact match can be found. An adolescent in the sample of relatively poor may for instance have 0.333 in propensity score, while among the non-poor, it could easily happen that no one has exactly this score, but fairly close scores are present, for instance in the 0.28–0.32 range or the 0.34–0.37 range. Which non-poor adolescents should be included as matches, and how should the matches be weighted?

Matching algorithms provide different techniques for handling these questions. In *Radius* matching, the selection of matches among the non-poor adolescents are restricted to those with propensity scores within a certain range, defined by a *caliper* value. Thus, if an adolescent exposed to relative poverty has propensity score 0.333, and the caliper value is set to 0.02, adolescents in the non-poor sample with propensity scores in the 0.313–0.353 range will be selected as matches. If no matches are found within the specified range, the exposed unit will be excluded from further analyses, because inclusion would threaten the comparability between the two samples. A small caliper value will result in very similar distributions of propensity scores which may improve the estimations of counterfactual effects. However, more sample units will be excluded which may threaten the validity of the estimations, and if the analyzed samples become too small, random errors may increase. A larger caliper value will, on the other hand, keep more sample units in the analyses, at the cost of more differences between the two samples in the distribution of propensity scores.

In our estimation by means of Radius matching, we used caliper value 0.02, which provides a fair balance between the number of cases kept in the analysis and the similarity in propensity score distributions.

In *Kernel* matching, the value stipulated for bandwidth serves the same purpose as the caliper in Radius matching, *i.e.*, designating what cases are to be included in the matching sample. When using Kernel matching, we chose bandwidth value 0.03 in order to check whether the pattern of results remained when more cases were included in the analysis. With Kernel matching each unit included in the matching sample is weighted in proportion to the distance in propensity scores between the exposed unit and the matching units.

Propensity score matching facilitates the estimation of whether effects differ between various subcategories. Here, two types of effects are estimated. The *Average treatment effect on the treated* refers, in this paper, to the estimated effect of relative poverty on subjective health in the sample of relatively poor (*i.e.*, those who actually experienced relative poverty during 2004–2008). Correspondingly, the *Average treatment effect on the untreated* refers to the hypothetical effect of relative poverty on the subjective health among those adolescents who were not poor during 2004–2008. These two effects may be different for several reasons. It could happen, for instance, that the average effect of relative poverty among the relatively poor is influenced by a higher exposure to deprivation in a more general sense. Conversely, the counterfactual effect of becoming relatively poor among those adolescents who actually were not poor, might be less severe if neutralized by a generally more favourable life situation. We have utilized the calculations of T-values in STATA *psmatch2* software in order to evaluate the statistical significance of the two effects.

In order to broaden the basis for interpretations, we will, as mentioned above, analyze adolescents’ subjective health not only by two different matching algorithms, but also by measuring subjective health in two ways; first, as the *level* of subjective health in 2009, and secondly, by taking advantage of the panel structure of the data and analyze *change* in subjective health from 2003 to 2009. The latter version has the advantage that it will approximate a removal of bias due to any stable unobserved characteristic that could have influenced subjective health at both time points.

## 4. Results

In the analyzed sample of 510 adolescents, the mean value on the subjective health index was 9.96 (standard deviation SD 5.73) in the 2009 interviews when the adolescents were aged 16–18. The average change from 2003 to 2009 was +2.30 (SD 6.64); thus, overall subjective health complaints increased from early to late adolescence. Gender differences were considerable (average level in 2009 was 7.66 for boys and 12.04 for girls). In this study, however, the two genders will be analyzed together in order to preserve the size of the analyzed sample. This will not influence the estimation of the effects of relative poverty, since the proportion of girls in the two samples is quite similar.

Table 1 shows the values for the outcome variables, separately for the relatively poor and the non-poor adolescents. Clearly, the relatively poor adolescents reported more subjective health complaints in 2009 than the non-poor adolescents (mean values 11.07 *versus* 9.66). When using the conventional *p* < 0.05 threshold, the difference (1.41) was statistically significant (*p* = 0.02). Also, subjective health worsened more on average from 2003 to 2009 among the relatively poor; the mean value increased by 3.70, compared to 1.92 among the non-poor, and also this difference was statistically significant (*p* = 0.01).

**Table 1:** Descriptive statistics of the sample of adolescents.

	Relatively poor	Not poor	*p*-value
	(N = 109)	(N = 401)	
**Adolescent subjective health**			
Mean index value 2009	11.07	9.66	0.022
Mean change 2003–2009	3.70	1.92	0.013
**Demographics**			
Girls (%)	55.1	51.9	0.557
Adolescents’ mean age in 2009	17.2	17.1	0.483
Living with both biological parents (%)	59.6	67.2	0.142
One biological and one step-parent (%)	6.4	10.0	0.172
One-parent family (%)	33.9	22.7	0.016
Mean age interviewed parent, 2009	43.5	46.0	0.011
Mean number of family members, 2003	5.03	4.56	0.004
Non-Western immigrant background (%)	30.3	7.2	<0.001
**Household finances 2000–2003**			
Mean household-adjusted income (NOK)	122.000	186.000	<0.001
Mean household-adjusted financial assets (NOK)	14.800	124.000	<0.001
**Parental characteristics**			
Father’s education, mean number of years	10.4	12.3	<0.001
Mother’s education, mean number of years	9.7	12.3	<0.001
Self-rated health very good, interviewed parent (%)	27.5	40.4	0.009
Self-rated health very good, parent’s partner (%)	22.0	32.2	0.103
Interviewed parent 3+ mental health complaints (%)	22.9	11.2	0.002
No adult in family employed 2003 (%)	32.1	8.0	<0.001
Low-income subsample in 2000 (%)	96.3	71.8	<0.001

Furthermore, Table 1 shows how the two samples differed with respect to the variables which were utilized in the estimation of propensity scores. The non-poor sample had experienced a better family economy before 2004, and their parents were mostly better educated than the parents of the relatively poor adolescents. The relatively poor adolescents had more often parents who were afflicted by health problems, they lived more often in one-parent families and/or in families with no employed adults, and they had more often a non-Western immigrant background.

Table 1 illustrates the difficulties which have to be dealt with when trying to establish whether relative poverty, *by itself*, contributes to the higher level of subjective ill health among the relatively poor adolescents. Not only was the economic situation in the families of the non-poor much better; the non-poor experienced more advantageous environments also regarding a series of other life circumstances, such as the parents’ educational level, employment, and health.

**Figure 1:**
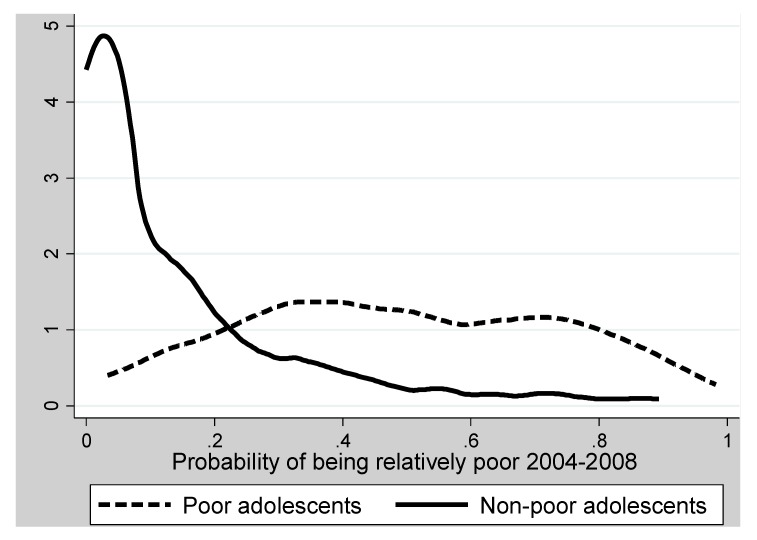
Density of propensity scores for the sample of relatively poor and the sample of non-poor adolescents.

The differences between the two samples are further demonstrated in Figure 1, which indicates how the estimated propensity scores, *i.e.*, the probability of being relatively poor, were distributed in the two samples. Among those who were relatively poor, the mean estimated propensity score was 0.490 (range 0.033 to 0.982); among the non-poor, the mean was 0.136 (range from <0.001 to 0.893). Thus, both samples are represented across a wide propensity score range, but the shapes of the distributions are drastically different. The non-poor had fairly many with extremely low probability of being poor, while a few of the relatively poor adolescents had a probability of being poor exceeding 0.9. Figure 1 illustrates the large disparities between the two samples in their probabilities of becoming relatively poor, and this underlines the challenge for causal analysis since the two samples were so dissimilar as to many life aspects which could influence their subjective health.

Nevertheless, Figure 1 also demonstrates that for most propensity scores levels, at least a few among the non-poor have propensity scores which are similar to the propensity scores of some of the relatively poor adolescents. There is therefore a considerable *common support area*, *i.e.*, in the probability range from 0.033 to 0.893, both poor and non-poor can be found, which allows for using the matching techniques. We utilize the two methods described above—Radius and Kernel matching—for estimating the counterfactual effects of relative poverty. The results are given in Table 2.

**Table 2:** Effects of relative poverty on subjective health among relatively poor adolescents (upper part) and non-poor adolescent (lower part), Radius and Kernel propensity score matching.

	Value if poor	Value if not poor	Effect	T-value	*p*-value
**Effects on relatively poor adolescents**					
*Subjective health in 2009, estimations by*					
—Radius matching, caliper 0.02	11.06	9.36	1.70	1.81	0.071
—Kernel matching, bandwidth 0.03	10.98	9.44	1.54	1.65	0.099
*Change in subjective health 2003–2009, estimations by*					
—Radius matching, caliper 0.02	3.83	1.25	2.59	2.40	0.017
—Kernel matching, bandwidth 0.03	3.58	1.43	2.15	2.00	0.046
**Effects on adolescent who were not poor**					
*Subjective health in 2009, estimations by*					
—Radius matching, caliper 0.02	11.22	9.92	1.30	0.84	0.401
—Kernel matching, bandwidth 0.03	11.42	9.94	1.48	0.76	0.448
*Change in subjective health 2003–2009, estimations by*					
—Radius matching, caliper 0.02	2.10	1.95	0.15	0.09	0.928
—Kernel matching, bandwidth 0.03	1.26	1.98	−0.72	−0.33	0.742

The number of adolescent respondents included in the estimations of counterfactual effects were 103 poor and 292 non-poor when using Radius matching caliper 0.02; and 107 poor and 339 non-poor when using Kernel matching with bandwidth 0.03. Estimations were made by STATA version 11.2, psmatch2 software.

The upper part of Table 2 shows the estimated counterfactual effects of relative poverty for the relatively poor adolescents; these estimations refer to what was termed the *Average treatment effect on the treated* in Section 3.4. Both Radius matching, caliper 0.02, and Kernel matching with bandwidth 0.03, indicate some effect of relative poverty on the 2009 level of subjective health (1.70 and 1.54, respectively). The *p*-values for both these estimates are somewhat higher than the conventional *p* < 0.05 threshold for statistical significance, however.

With respect to *change* (*i.e.*, deterioration in subjective health) from age 10–12 in 2003 to age 16–18 in 2009, the effects are more marked: 2.59 when using the Radius technique and 2.15 when using the Kernel technique. Both estimates have *p*-values below 0.05. Accordingly, these estimations indicate that living in relative poverty among these adolescent categories had a detrimental impact on the development of subjective health during adolescence.

The estimated counterfactual effects of relative poverty among those adolescents who did not experience poverty during 2004–2008—termed the *Average treatment effect on the untreated* in section 3.4—are clearly weaker (lower part of Table 2). The size of the effect on the 2009 level of subjective health does not seem negligible at first sight (1.30 with Radius matching, 1.48 with Kernel matching), but they are far from statistically significant.

Regarding change in subjective health during adolescence among the non-poor adolescents, the estimated effects (close to zero with Radius matching, slightly *negative* with Kernel matching) indicate that for this category of adolescents, the impact of a hypothetical exposure to relative poverty would have been practically nil.

## 5. Discussion

### 5.1. Main Results and Interpretation

This study has asked whether relatively poverty in contemporary Norwegian society is detrimental for adolescents’ subjective health, which was measured by their reporting of somatic and mental symptoms. The findings indicate that for adolescents who lived in relatively poor families, the economic situation in their families had a negative effect on their subjective health trajectory during adolescence from age 10–12 to age 16–18. Also the results regarding the level of subjective health when these adolescents were 16–18 years old, suggest a similar conclusion about some negative effect of limited economic resources in the adolescents’ families, although this latter effect was weaker.

This study has employed statistical techniques which aim at neutralizing the confounding influence from other factors than the assumed cause. Caution must always be applied when drawing causal conclusions from observational studies. Nevertheless, the results are at least compatible with the hypothesis that among the adolescents who lived in relatively poor families, the restricted economic resources in these families contributed to the causal processes which led up to their less favourable subjective health situation in late adolescence.

This conclusion can only be drawn for those adolescents who actually lived in relative poverty, however. For those adolescents who lived in families with economic resources above the relative poverty level, the counterfactual modelling utilized in this study could not reveal any potential negative health effect in the hypothetical case that these families had been exposed to relative poverty.

Thus, the findings point towards a differentiated conclusion: For those adolescents who actually experienced relative poverty, their economic situation aggravated their subjective ill health; for those adolescents who were not exposed to relative poverty, it is not likely that their subjective health would have worsened if they actually had lived in relative poverty.

A possible interpretation of this empirical pattern could take into consideration that relatively poor adolescents are often experiencing other disadvantageous circumstances apart from their families’ economic situation. They are, for instance, more often living in single-parent families, they have often less educated parents, or parents who are out of work, or parents afflicted by health problems (Table 1). The non-poor adolescents are less affected by such circumstances, which could mean that there are more protective factors in their environments which buffer against the potential detrimental health consequences of few economic resources in the family. Among the relatively poor adolescents, on the other hand, who more often lack such protective environments, relative poverty could add to their other disadvantages in a way which leads to deteriorated subjective health. Whether this occurs in line with the Family Economic Stress Model or the Investment Model [5,7] as discussed in the introduction, or through some other type of process, cannot be further pursued in this paper.

How important are these effects on the subjective health of relatively poor adolescents? Certainly, an array of factors contribute to young people’s subjective health. A family’s economic situation, in the context of the contemporary Norwegian welfare state, is only one of many factors which could contribute to adolescents’ symptom burden. It should nevertheless be noted that the results of this study suggest that if the relatively poor adolescents had lived in non-poor families, their subjective health development during adolescence would not deviate, on average, from that experienced by other adolescents. The observed difference between relatively poor and non-poor adolescents with regard to the deterioration in subjective health 2003–2009 (Table 1) was smaller than the effects for the relatively poor adolescent estimated by propensity score matching (Table 2). The detrimental effect of relative poverty detected in this study seems therefore, in statistical terms, to account for the difference between relatively poor and non-poor in how subjective health developed during adolescence.

### 5.2. Strengths and Limitations

The data available for this study have several advantages. Information about subjective health has been obtained in personal interviews—not with proxy respondents, but with the adolescents themselves. Various other information about the adolescents’ situation was collected from one of the parents, while the crucial independent variable in this study, families’ economic resources, was constructed on the basis of register data which probably are more objective and valid than survey answers. The data were furthermore obtained from a prospective panel design, enabling a fairly certain time ordering of variables. Thus, these data avoid some of the sources of error which have plagued many youth studies [28]. Moreover, the causal analyses have utilized the techniques of propensity score matching, which arguably is a promising tool for social science when the purpose is to approximate estimations of causal effects in observational data [34].

A critical aspect of propensity score matching is the procedure for estimating propensity scores, *i.e.*, the calculation of the probability of being assigned to the category exposed to the assumed cause. This study has had access to rich information which could imply that the estimated probabilities do not deviate dramatically from the true propensity scores. However, an inclusion of more predictors in this calculation—for instance variables indicating parents’ personal characteristics, their social skills, and their previous working career—could certainly have improved the estimation of the propensity scores.

Various limitations are also evident. Although the adolescents’ families represent many levels of economic circumstances, we cannot reject the possibility that the self-selection of respondents which occurs in interview surveys has resulted in a sample bias which could influence the results. Sample attrition has been substantial, and the analyse have been performed on a sample which is restricted in size. This is clearly a source of uncertainty. Moreover, the results from the two utilized propensity score matching techniques are not identical. Deviating results may occur in small samples because the techniques vary in terms of what units are included in the matching samples. The basic similarity in the results from the two matching techniques gives however some credibility to the main findings. Still, the propensity score matching technique cannot overcome the basic problem affecting all causal analyses based on observational studies: The potential impact of unknown, unobserved, variables.

Generalizing the results of the present study to the situation in other countries would imply assumptions which are difficult to assess. The Norwegian case, with its special economic, political, and cultural situation, could be special. Similar studies in other settings are needed in order to examine whether the effects on relatively poor adolescents’ subjective health which were detected in this study, correspond to similar effects in other countries.

## 6. Conclusions

Living in families with a limited access to economic resources, in the contemporary Norwegian social context, appears to have detrimental effects on the development of subjective health during adolescence among those adolescents who are exposed to relative poverty. In families with more economic resources, it is likely that better access to other advantageous environments buffers against the potentially detrimental health effects of relative poverty. The results indicate that inequalities in families’ economic resources contribute to the health inequalities among Norwegian adolescents.
